# Inflammatory Regulation in Diabetes and Metabolic Dysfunction

**DOI:** 10.1155/2017/5165268

**Published:** 2017-03-15

**Authors:** Jixin Zhong, Quan Gong, Akira Mima

**Affiliations:** ^1^Department of Endocrinology, Central Hospital of Wuhan, Wuhan, Hubei 430061, China; ^2^Cardiovascular Research Institute, Case Western Reserve University, Cleveland, OH 44106, USA; ^3^Department of Immunology, School of Medicine, Yangtze University, Jingzhou, Hubei 434023, China; ^4^Department of Nephrology, Kindai University Nara Hospital, Faculty of Medicine, Kindai University, Nara 630-0293, Japan

The research in the past decades has revealed a critical link between metabolic disorders and inflammation, which leads to a concept called “metaflammation.” Metaflammation is a form of low-grade systemic and chronic inflammation related to excess nutrients and energy [1, 2]. There has been increasing evidence showing diabetes is an inflammatory disease. Type 1 diabetes (T1DM), characterized by autoimmune-mediated destruction of pancreatic *β* cells and insufficient secretion of insulin, has long been considered as an inflammatory disease. Not until the early 1990s, however, was type 2 diabetes (T2DM) linked to inflammatory response [3]. T2DM is manifested by peripheral insulin resistance and aberrant production of insulin, accompanied by chronic low grade inflammation in peripheral tissues such as adipose tissue, liver, and muscle. In the last decades, there has been growing evidence linking obesity and insulin resistance to inflammation [2–4]. Given the significant roles inflammation plays in its pathogenesis, T2DM is now being redefined as an immune disorder [5–7]. In addition to diabetes, many other metabolic disorders have also been associated with inflammation [2]. This special issue showcases a number original research articles and review papers on the topic of inflammatory regulation in metabolic dysfunction.

Animal models are useful tools to study pathogenic mechanisms including inflammation in diabetes. In this special issue, Y. Takeda et al. introduced a number of rat models of diabetes and discussed the immune features in those diabetic rat models. A better understanding of the immune responses in different diabetes models may help the researchers choose an appropriate model in their studies.

Innate immune response has long been recognized to play an essential role in the development of diabetes and metabolic regulation [2]. Y. I. Sánchez-Zamora et al. reported a novel role for macrophage migration inhibitory factor (Mif) in regulating macrophage/dendritic cell response and T1DM. Using a STZ-induced diabetes model and Mif knockout mice, their study suggested that Mif upregulates the expressions of MHC-II, costimulatory molecules CD80, CD86, and CD40, Toll-like receptor- (TLR-) 2, and TLR-4 and thus promotes the activation of macrophage/dendritic cell response and Th1 response in T1DM. J. Juwono and R. D. Martinus reviewed the role of heat shock protein 60 (Hsp60), a mitochondrial stress protein, in the pathogenesis of T1DM and T2DM. Y. Wang et al. summarized the role of HMGB1, an endogenous danger signal molecule in the development of T2DM. In recent years, the role of adaptive immunity in T2DM has also been emphasized [4, 8–10]. In this issue, C. Xia et al. reviewed the recent progress in understanding the role of T lymphocyte-mediated adaptive immune response in the inflammatory regulation of T2DM.

Cytokines are important regulators of inflammation in a number of disease conditions. Z. Wang et al. examined the inflammatory markers in prediabetes by analyzing 215 patients with prediabetes, 126 cases of newly diagnosed T2DM, and 219 controls with normal glucose tolerance. Plasma Hs-CRP, IgE, IL-4, IL-10, and tryptase levels were identified to increase in prediabetes and early T2DM. C. S. Nunemaker addressed the differences in the inflammatory cytokines from the perspective of the pancreatic islet in type 1 versus type 2 diabetes and proposed to consider alternative models of cytokine exposure to reflect the pancreatic environment in T2DM more accurately. H. Jin et al. demonstrated in an original research article that increased IL-6 level in collagen-induced arthritis was associated with elevated fasting blood glucose and reduced fasting insulin level. This may be caused by IL-6-induced activation of caspase-3 and loss of pancreatic islet cell. L. Duan et al. reported an association between serum IL-33 and lipid metabolism dysfunction, a common reason for kidney injury in gout. In addition, IL-33 was higher in gout patients without kidney injury compared to patients with kidney injury, suggesting a potential role of IL-33 in lipid metabolism and kidney injury in gout patients.

Collectively, all research papers and review articles in this special issue highlight the current status of research in the area of inflammatory regulation in metabolic disorders. Despite the rapid growth in this area, we still have a long way to go to fully understand the inflammatory mechanisms of metabolic disorders.
